# Wide-Band Spatially Tunable Photonic Bandgap in Visible Spectral Range and Laser based on a Polymer Stabilized Blue Phase

**DOI:** 10.1038/srep30407

**Published:** 2016-07-26

**Authors:** Jia-De Lin, Tsai-Yen Wang, Ting-Shan Mo, Shuan-Yu Huang, Chia-Rong Lee

**Affiliations:** 1 National Cheng Kung University, Department of Photonics, Tainan, 701, Taiwan; 2 Kun Shan University of Technology, Department of Electro-Optical Engineering, Tainan, 710, Taiwan; 3 Chung Shan Medical University, Department of Optometry, Taichung, 402, Taiwan; 4 Chung Shan Medical University Hospital, Department of Ophthalmology, Taichung, 402, Taiwan; 5 National Cheng Kung University, Advanced Optoelectronics Technology Center, Tainan, 701, Taiwan

## Abstract

This work successfully develops a largely-gradient-pitched polymer-stabilized blue phase (PSBP) photonic bandgap (PBG) device with a wide-band spatial tunability in nearly entire visible region within a wide blue phase (BP) temperature range including room temperature. The device is fabricated based on the reverse diffusion of two injected BP-monomer mixtures with a low and a high chiral concentrations and afterwards through UV-curing. This gradient-pitched PSBP can show a rainbow-like reflection appearance in which the peak wavelength of the PBG can be spatially tuned from the blue to the red regions at room temperature. The total tuning spectral range for the cell is as broad as 165 nm and covers almost the entire visible region. Based on the gradient-pitched PSBP, a spatially tunable laser is also demonstrated in this work. The temperature sensitivity of the lasing wavelength for the laser is negatively linear and approximately −0.26 nm/°C. The two devices have a great potential for use in applications of photonic devices and displays because of their multiple advantages, such as wide-band tunability, wide operated temperature range, high stability and reliability, no issue of hysteresis, no need of external controlling sources, and not slow tuning speed (mechanically).

In recent years, spatially micro/nanostructured materials have attracted increasing interest in a wide range of applications in many fields, such as optical communication^1^, microelectronics^2^, and biological detection^3^. However, their complex fabrication remains a significant challenge despite the improvement in nanotechnology^4^. Fortunately, an unusual liquid crystal (LC) material, blue phase (BP), can self-assemble as a 3D photonic crystal (PhC)-like micro/nanostructure without any complicated processing. BPs exhibit the selective Bragg reflection in the visible region because of their periodic micro/nanostructures with lattice constants of several hundred nanometers^5^. Regarding their applications, BPs are promising for use in fast light modulators^6^,^7^ or tunable photonic bandgap (PBG) and filtering devices^8^,^9^,^10^,^11^. In particular, emission tuning and lasing action can provide new applications for high-Q resonators or low-threshold lasers based on small optically active PhC-like BP devices^12^,^13^,^14^,^15^. However, the narrow BP temperature range (about a few K) limits their realistic applications. This issue was solved by employing the polymer-stabilized method, which successfully extends the BP temperature range over 60 °C^16^. The polymer-stabilized BP (PSBP) device can even work in BP at room temperature without a cumbersome temperature-controlling system. This advantage enables BPs to be successfully used in real applications of photonic devices and displays^17^,^18^,^19^,^20^. LCs are widely known for their highly flexible tunability using various methods, such as applying voltage and stress, heating/cooling, illumination by actinic light, and changing pumped position. However, under the stabilization of the polymer network in the disclination lines, the BP lattices are usually steady or less changed in the presence of external stimuli. Therefore, tens of volts is unavoidably necessary to be applied on the PSBPs for electrically tuning their PBGs and the tunable wavelength range is limited or discontinuous^21^,^22^,^23^. Even though the DC-field driving method can significantly improve the tuning range of the PBG of PSBP by trapping the ions within it and elongates the lattice constant^24^, response time and stability are two other important issues and should be also concerned. The spatial tuning approach is the preferable method enabling the maintenance of the tuning feature of the BP device and high stability (wide BP temperature range) after polymer stabilization.

As a result of the motives mentioned above, we fabricate and demonstrate a linearly-gradient-pitched PSBP PBG device with a widely-spatial tunability based on the reversed diffusion of two injected BP mixtures with low and high chiral concentrations and then UV irradiation in this study. Experimental results indicate that the formed PSBP PBG device can be tuned spatially from blue (481.9 nm) to red (646.9 nm) regions within 14 mm at room temperature and the total tuning band is as wide as 165 nm. This work also develops a polymer-stabilized dye-doped BP (PSDDBP) laser and discusses associated lasing features and wavelength tunability. Experimental results show that the tunable band of the laser is 57.7 nm (from 552.9 nm to 610.6 nm) at room temperature, which is 82 nm narrower than that of the corresponding PBG. This narrowing is primarily determined by several factors, such as the dye’s reabsorption of fluorescence photons at short wavelength regions, weak dye’s fluorescence emission at long wavelength regions, and significant fragmentation of the frustrated BP structure at long wavelength regions. The temperature-dependence of the lasing wavelength for the PSDDBP laser is linear, and the associated temperature sensitivity is approximately 0.26 nm/°C. Such temperature sensitivity is mainly attributed to the constant and negative *dn/dT* of the LCs. Given the advantages of the PBG and laser devices, they have great potential for use in applications of photonic devices and displays.

## Results

### Spatially-tunable photonic bandgap of a gradient-pitched polymer-stabilized blue phase

**Figure 1:**
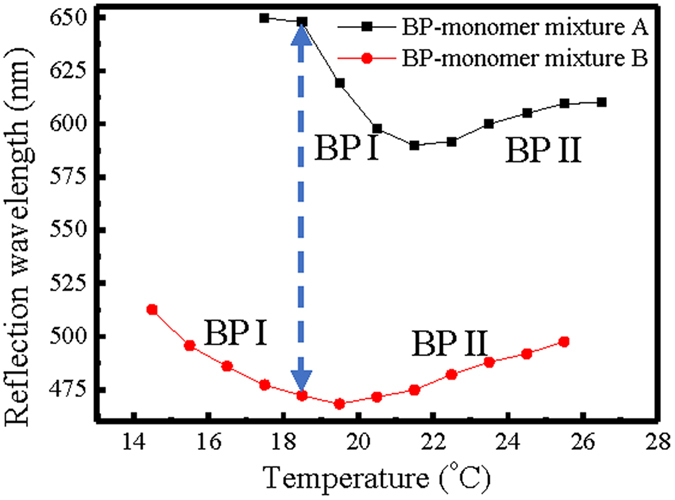
Temperature dependences of the peak reflection wavelength for cells with BP-monomer mixtures A and B. The black and red curves with square and circle dots, respectively, present the temperature-dependent peak reflection wavelength of BP-monomer mixtures A and B, respectively.

In this study, we inject two BP-monomer mixtures A and B into an empty cell to obtain a gradient-pitched PSBP sample (see Table 1 for the recipes and Methods). Before examining the properties of the gradient-pitched PSPB sample, the BP temperature ranges for the BP-monomer mixtures A and B which are filled in two identical empty cells with 15-μm-thickness should be confirmed previously. This can be done by the observation of the appearance of the LC texture and the measure of the reflection spectra under the reflective polarizing optical microscope (R-POM). The two cells with mixtures A and B are both elevated to isotropic state (*T* = 30.5 °C) and then cooled down slowly at a fixed rate of 0.04 °C/min. Figure 1 shows the variations in the peak wavelength of the PBG with the temperature for the two cells filled with mixtures A and B. Apparently, the BP temperature ranges for the cells with mixtures A and B are between 26.5 °C and 17.5 °C and between 25.5 °C and 14.5 °C, respectively. The peak reflection wavelength of the cell with mixture A or B initially blue-shifts and then red-shifts during the cooling process. The opposite shift of the reflection wavelength in the course of decreasing temperature based on the BP-monomer system implies the phase transition from BP II to BP I, which result can be identified by the transition of the LC texture from BP II to BP I observed under the R-POM (displayed in Supplementary Fig. S1), which result is similar to that based on a pure BP system^25^. However, the sole difference between the temperature-dependent optical properties of the two systems is its continuity (or discontinuity) at the transition temperature. The variation of the reflection wavelength at the transition temperature is discontinuous for a pure BP system, but continuous for the BP-monomer system A or B used in the present study (Fig. 1). The phenomenon for the continuous change in the peak reflection wavelength with changing temperature in the BP-monomer systems is also observed in a nanoparticle (NP)-doped BP system^26^. The NPs or the monomers used in the present work seems to be able to stabilize the BP structure so as to avoid the abrupt phase change between BP II and BP I.

**Figure 2:**
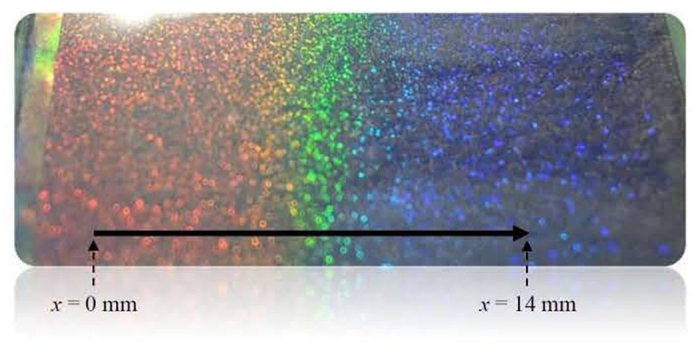
Reflective appearance of the formed gradient-pitched PSBP sample.

**Figure 3:**
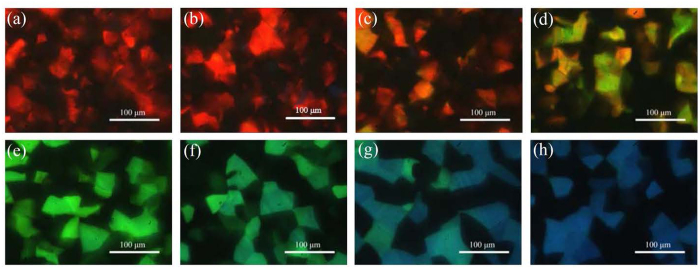
(**a–h**) R-POM images of the gradient-pitched PSBP sample at *x* = 0, 2, 4, 6, 8, 10, 12, and 14 mm, respectively. The scale bar is 100 μm.

**Figure 4:**
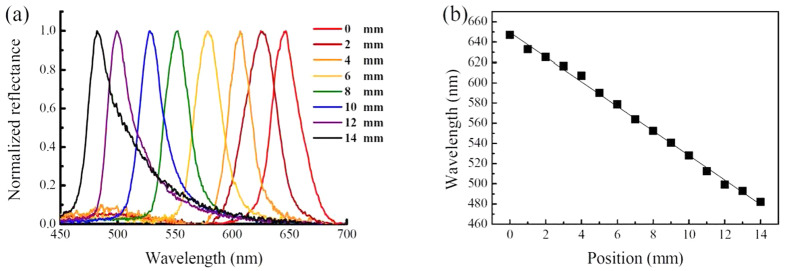
Spatially tunable PBG of the gradient-pitched PSBP. (**a**) Reflection spectra of the gradient-pitched PSBP measured at *x* = 0, 2, 4, 6, 8, 10, 12, and 14 mm, with peak wavelengths of 646.9, 625.1, 607.1, 578.8, 552.5, 528.2, 499.3, and 481.9 nm, respectively. (**b**) Variation of the peak wavelength of the PBG for the gradient-pitched PSBP cell with position. The spatial gradient d*λ*_*p*_/dx is 12.15 nm/mm.

As presented in Fig. 1, the largest difference in reflection wavelengths (from 472.19 nm to 647.84 nm) of cells with BP-monomer mixtures A and B can be achieved at 18.5 °C. Therefore, 18.5 °C is the optimal temperature for curing the BP-monomer cell with largest pitch gradient. Following the fabrication process described in Methods, a gradient-pitched PSBP sample with a large gradient of reflective color ranging from red to blue regions at positions *x* = 0 mm to *x* = 14 mm at room temperature can be fabricated. Figure 2 shows the appearance of the formed PSBP sample with a large pitch gradient. The gradient-pitched PSBP cell shows a rainbow-like reflection pattern at positions *x* = 0 mm to *x* = 14 mm. Figures 3(a–h) show the R-POM images of the gradient-pitched PSBP cell at *x* = 0, 2, 4, 6, 8, 10, 12, and 14 mm, respectively, at room temperature. As shown in Fig. 3(a–h), the color of the BP micro-platelets in the gradient-pitched PSBP cell varies gradually from red to blue at *x* = 0, 2, 4, 6, 8, 10, 12, and 14 mm, respectively. These micro-platelets in various regions can reflect selectively the incident light such that a grain-like reflective appearance with various colors is observed for the gradient-pitched PSBP cell, as displayed in Fig. 2. The corresponding reflection spectra of the cell at *x* = 0 mm to *x* = 14 mm are also measured at room temperature and presented in Fig. 4(a). Apparently, the PBG of the cell is continuously distributed from the red to blue regions at positions *x* = 0 mm to *x* = 14 mm, with PBG peaks at wavelengths of *λ*_*p*_ = 481.9 nm to *λ*_*p*_ = 646.9 nm. The total tuning spectral range for the gradient-pitched PSBP cell is as broad as 165 nm and covers almost the entire visible region. Figure 4(b) shows the variation in the peak wavelength of the PBG of the gradient-pitched PSBP cell with position. The variation is negatively linear, that is, *dλ*_*p*_*/dx* is negative and constant (in the present case, *dλ*_*p*_*/dx* = 12.15 nm/mm). A constant *dλ*_*p*_*/dx* is convenient to use for applications such as the spatially tunable PBG devices.

The phase and the crystal structures of the gradient-pitched PSBP cell at various positions can be identified by examining the corresponding Kossel diagrams^27^ at those positions. However, the gradient-pitched cell is not suitable for use in measuring Kossel diagrams for several reasons. As described in Methods, to efficiently observe the Kossel diagram of the BP cell under an optical microscope, a high magnification oil immersion objective with a high NA value is needed. However, this objective has a short working distance. To increase the operation space of the objective, the cell should be made using thin coverslip. In addition, the pitch should be uniform on the detected area of the cell when measuring the Kossel diagrams. Non-uniform pitch causes non-uniform lattice on the detected region, making the observation of Kossel diagrams difficult. Hence, to obtain the Kossel diagrams of the gradient-pitched PSBP cell at various positions, six uniformly thick BP-monomer cells (with identical cell gap of 15 μm) with various chiral concentrations are prepared. The measured Kossel diagrams of these cells can be used to model the corresponding reflection features and crystal structures at various positions of the gradient-pitched PSBP cell. To achieve this purpose, the prescriptions of the six BP-monomer cells are deliberately arranged, as shown in Supplementary Table S1. The six cells are made by filling six BP-monomer mixtures, marked by BP-monomer mixtures A, C, D, E, F, and B in order, into six identical empty cells. Each empty cell is pre-fabricated by combining one ITO glass and one thin cover glass slide with 15-μm-thick spacers between them. The six BP-monomer mixture cells are heated to the isotropic phase and then cooled down at a cooling rate of 0.04 °C/min. The cells with BP-monomer mixtures A, C, D, E, F, and B exhibit a gradually decreasing variation in the reflection peak wavelength from 647.84 nm to 472.19 nm at the curing temperature of 18.5 °C, as shown in Supplementary Table S2 and Fig. S2. After identical UV irradiation of 1.1 mW/cm^2^ for 30 min at 18.5 °C, each cell can become PSBP with very small changes in the reflection peak of the PBG. The cells filled with mixtures A, C, D, E, F, and B after UV irradiation are referred to as PSBP A, C, D, E, F, and B, respectively. Compared with the results displayed in Fig. 4(b), the linear variation in the reflection peak wavelength with the chiral concentration measured at room temperature (Supplementary Fig. S2) is consistent with that with the position of the gradient-pitched PSBP cell. Therefore, the PSBP A, C, D, E, F, and B can be used to equivalently find out the corresponding Kossel diagrams at positions *x* = 0, 2.8, 5.6, 8.4, 11.2, and 14 mm of the gradient-pitched PSBP cell, respectively, where the reflection peak wavelengths are similar to those for the six PSBP cells.

**Figure 5:**
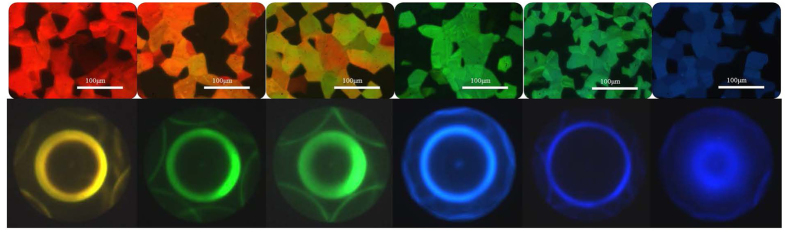
R-POM images and Kossel diagrams of PSBP cells. (Upper row, from left to right) R-POM images of PSBP cells A, C, D, E, F, and B, respectively. The normal reflection peak wavelength of the PBG in the cells is *λ*_*h,k,l*(*norm.*)_ = 640, 608, 576, 540, 512, and 470 nm. Respectively. The scale bar in each sub-figure is 100 μm. (Bottom row, from left to right) The corresponding Kossel diagrams measured when the wavelength of the monochromatic light for each Kossel diagram is selected as 580, 550, 535, 488, 456, and 456 nm, respectively.

The lattice structures of the PSBP cells A, C, D, E, F, and B can be determined based on the corresponding Kossel diagrams. For a clear image of the Kossel diagram with less background noise, the cooling rate is set to as slow as 0.01 °C/min to obtain large platelets in the PSBP cells. Figure 5 (from the left- to the right) shows the R-POM images (upper row) and the corresponding Kossel diagrams (bottom row) of PSBP cells A, C, D, E, F, and B in order using probe beams with various wavelengths. As described by the previous literature^27^, to obtain a Kossel diagram of specific BP lattice planes (*h, k, l*), the incident wavelength of a monochromatic probe beam, *λ*, has to be shorter than the Bragg wavelength of normal reflecting light of these specific lattice planes, *λ*_*h,k,l*(*norm.*)_, that is, *λ*_*h,k,l*(*norm.*)_ > *λ*. The upper row in Fig. 5 shows the red, orange, orange-green, green, green-blue, and blue BP platelets in the PSBP cells A, C, D, E, F, and B, respectively, observed under the R-POM, with the normal reflecting peak wavelengths of 640, 608, 576, 540, 512, and 470 nm, respectively. Clear diffraction patterns can be obtained and those displayed in the bottom row of Fig. 5 are selected representatively for which the wavelength of the incident light is shorter than the peak wavelength of the reflection (All diffraction patterns are provided in Supplementary Figs S3–S8). As shown in Fig. 5, all PSBP cells exhibit Kossel lines with a bright circle in the center and two pairs of curves with twofold symmetry surrounding the centered circle. The bright circle in the Kossel lines is caused by diffraction of the reflecting plane set of (110) of BP I observed in the viewing direction of (110). The twofold symmetric curves are formed by diffraction of other oblique reflecting planes with respect to the viewing direction of (110)^27^,^28^. By contrast, no effective diffraction pattern can be observed if the condition of *λ*_*h,k,l*(*norm.*)_ > *λ* is not satisfied, as demonstrated in Supplementary Figs S3–S8.

The gradient-pitched PSBP cell fabricated in this work can be applied as a wide-band spatially tunable PBG device with a high stability. The PBG of the PSBP hardly changes with tuning temperature or by applying an external electric or optic field because of the strong stability of the BP lattice by the polymer network^21^,^22^. Generally, the polymer of the BP structure is stable but loses its tunability, which is among the most attractive features of typical LC materials. Based on the present fabrication method shown in this work, the obtained PSBP cell with a large pitch gradient can gain strong stability but maintain its tunability within a wide spectral range via simply changing the detected position. In addition, the gradient-pitched PSBP has other advantages, such as wide operated temperature range including room temperature, not slow tuning speed (mechanically), no issue of hysteresis, and high reliability and stability. The gradient-pitched PSBP PBG cell can be used for other high-level applications, such as PSBP lasers with a high stability and tunability. Associated results about the lasing experiments will be described in the next section.

### Spatially-tunable polymer-stabilized dye-doped blue phase laser

**Figure 6:**
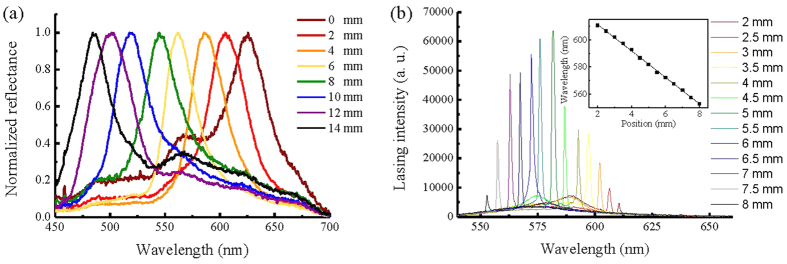
Reflection and lasing spectra of the gradient-pitched PSDDBP sample. (**a**) Reflection spectra measured at *x* = 0, 2, 4, 6, 8, 10, 12, 14 mm, with peak wavelengths of 625.3, 604.9, 586.1, 562.0, 544.7, 519.5, 500.2, 485.7 nm, respectively. (**b**) Obtained spectra of the lasing emission from the gradient-pitched PSDDBP cell pumped at *x* = 2 mm to *x* = 8 mm. The pumped energy is 13.0 μJ/pulse. Inset: Linear variation of the lasing wavelength of the gradient-pitched PSDDBP laser with the pumped position. The wavelength gradient of lasing emission is 9.77 nm/mm.

A gradient-pitched PSDDBP cell is also fabricated by doping laser dye into BP-monomer mixtures for performing the spatial tuning of the lasing emission in the present work. The laser dye doped in the PSBP laser is the pyrromethene dye (P597), which is considered a good candidate for dye lasers because of its low triplet‒triplet absorption capacity, poor tendency to self-aggregate, and thus fluorescence quenching, as well as high photostability^29^. The preparation of the gradient-pitched PSDDBP cell can be found in Methods. The PBG and lasing measurements of the cell are performed at room temperature (22.5 °C). Similar to the gradient-pitched PSBP cell, the gradient-pitched PSDDBP cell also shows a rainbow-like reflection pattern from *x* = 0 mm to 14 mm (see Supplementary Figs S9 and S10 for the appearance and R-POM images of the sample, respectively). The reflection spectra of the cell at *x* = 0 mm to *x* = 14 mm are measured and presented in Fig. 6(a). The PBG of the cell is extensively distributed from red to blue regions at positions *x* = 0 mm to 14 mm, with a PBG peak shifting from *λ*_*p*_ = 485.7 nm to *λ*_*p*_ = 625.3 nm. This total tuning spectral range for the gradient-pitched PSDDBP cell is also as broad as 140 nm. To examine the spatial tunability of the gradient-pitched PSDDBP laser, the cell is excited at positions *x* = 0 mm to *x* = 14 mm by the pumped pulses with a fixed energy of *E* = 13.0 μJ/pulse at room temperature. Figure 6(b) shows the obtained lasing signals with decreasing lasing wavelength from *λ*_*lasing*_ = 610.6 nm to *λ*_*lasing*_ = 552.9 nm pumped at *x* = 2 mm to 8 mm with a pumped step of 0.5 mm. In contrast to the PBGs displayed in Fig. 6(a), the lasing peaks all occur at the long wavelength edges of the corresponding PBGs at these positions. No lasing signal can be observed pumped at other positions (that is, *x* < 2 mm and *x* > 8 mm). The tunable spectral range of the laser is 57.7 nm, which is approximately 82.0 nm narrower than that of the PBG of the same sample. This result is primarily attributed to the following factors: a strong dye-reabsorption of the fluorescence photons below 550 nm at *x* > 8 mm and weak dye-fluorescence above 612 nm and significantly frustrated structure of BP at *x* < 2 mm. These factors lead to high energy thresholds that exceed the pumped energy to inhibit the lasing emissions at *x* > 8 mm and *x* < 2 mm. The inset in Fig. 6(b) shows the linear relation between the lasing wavelength and pumped position. This linearity increases the ease for spatially tuning the laser. To the best of our knowledge, this is the first tunable BP laser which can be operated at room temperature.

Photobleaching is a dynamic process in which fluorescence dye molecules lose their ability to fluoresce because of photo-induced chemical destruction upon exposure to excitation light. Moreover, photobleaching is the cumulative effect of loss fluorescence over time, and the rate of photobleaching is a function of the excitation intensity^30^. The pulse laser excitation with irradiated duration is roughly 20 s each time in the present lasing experiment. The diameter of the focused spot of the pulse laser, average energy per pulse, repetition rate, and pulse duration for excitation of the lasing emission of the PSDDBP are approximately 200 μm, 13 μJ/pulse [used to generate the lasing emission shown in Fig. 6(b)], 10 Hz, and 8 ns, respectively. The peak irradiation intensity at the sample can be estimated to be 13 μJ/[8 ns × π × (100 μm)^2^]≈5.2 MW/cm^2^. Under this condition, we did not observe apparent decay of the lasing emission, which indicates that no photobleaching of dye had occurred. Previous reports about solid-state dye lasers with P597 demonstrated that the service life of such lasers can be 60000 pulses at 25 MW/cm^2^ (5 Hz repetition rate)^31^ and over 10^6^ pulses at 1 mJ/pulse (20 Hz repetition rate)^32^,^33^. The peak irradiation intensity or the pumped energy of the pulse employed in the current work is lower than the pumped conditions shown in refs 31, 32, 33 to prevent photobleaching of dye.

**Figure 7:**
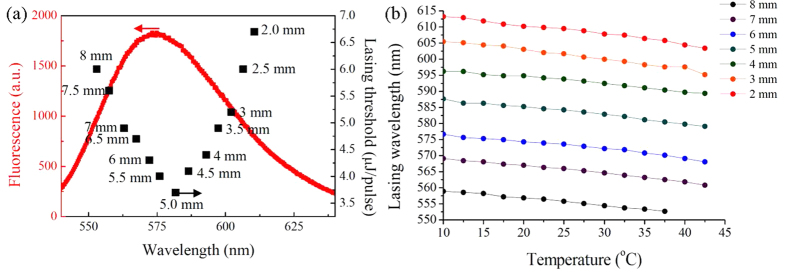
Lasing threshold and temperature-dependence of the spatially-tunable PSDDBP laser. (**a**) Variation of the energy threshold of the PSDDBP laser with the lasing wavelength and pumped position (represented by the full square dots). The fluorescence emission spectrum of the laser dye is also presented for reference (represented by the red curve). (**b**) Variation of the lasing wavelength of the gradient-pitched PSDDBP laser with temperature at *x* = 2 mm–8 mm. The average thermal sensitivity of the lasing wavelength is 0.26 nm/°C.

The energy threshold of the lasing emission can be found to be the pumped energy above which the lasing strength and the full-width at half-maximum (FWHM) abruptly rises and narrows, respectively. The details for the lasing features of the gradient-pitched PSBP laser at various pumped positions are displayed in Supplementary Fig. S11. The laser linewidth of the laser can be as narrow as 0.69 nm, which is similar to the optical resolution of the spectrometer used in this work. The variations in the lasing threshold of the laser with the lasing wavelength (or the corresponding pumped position) are indicated by the full square dots in Fig. 7(a). The fluorescence emission spectral curves of the laser dye (red curve) are also appended for discussion. The lasing threshold concaves upward as the lasing wavelength increases (or the pumped position decreases). As mentioned above, the dominant factors that determine the lasing threshold primarily include the strength of the dye’s fluorescence emission, dye’s reabsorption of the fluorescence photons within the absorption region, and the frustrated structure of the BP. At *x *≥ 5.0 mm, the lasing threshold increases with decreasing pumped position from 5.0 mm to 2.0 mm and increasing lasing wavelength. This phenomenon is attributed to both the decreasing strength of the fluorescence emission and increasing fragmentation in the frustrated BP structure with decreasing pumped position from 2.0 mm to 5.0 mm and zero reabsorption effect^34^. However, at *x* ≤ 5.0 mm, the lasing emissions occur near and within the absorption region. The increasing reabsorption of the fluorescence photons by the laser dyes may increase the lasing threshold through an increase in the pumped position from 5.0 mm to 8.0 mm. Figure 7(b) shows the temperature-dependence of the lasing wavelength of the gradient-pitched PSDDBP laser at *x* = 2 mm to 8 mm from 10 °C to 42.5 °C. Each curve is nearly linear and the slopes are very similar. An average thermal sensitivity of lasing wavelength (average slope) is approximately −0.26 nm/°C. The lattice constant of the BP in the PSDDBP cell at a specific position should keep constant with increasing temperature because of the strong anchor of the polymer network in the regions of disclination lines on the LCs in the BP lattices. The consistent decrease in lasing wavelength with increasing temperature measured at all positions must be caused by other factors that are less dependent on the lattice constant, such as the effective refractive index of the BP. The Bragg condition for the reflection of the cubic BP is shown in equation (1):


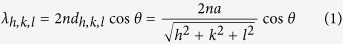


where *n* and *a* are the effective refractive index and lattice constant for a BP, respectively. The effective refractive index for a BP is equivalent to the average refractive index of the nematics organizing that BP. For most nematics, the average index is negatively linear to the temperature, that is, *dn/dT* is constant and negative^35^. This relationship leads to a constant and negative *dλ*_*h,k,l*_*/dT* because other factors in equation (1) remain unchanged. Suppose, reasonably, that *dλ*_*h,k,l*_*/dT* is nearly equal to *dλ*_*lasing*_*/dT* at each position. One can conclude that the constant and negative *dλ*_*lasing*_*/dT* shown in Fig. 7(b) can be obtained at each position because of the constant and negative *dn/dT* at each position.

## Discussion

This study successfully develops a gradient-pitched PSBP PBG device with a widely spatial tunability based on the reversed diffusion method of two injected BP-monomer mixtures with low and high chiral concentrations and UV irradiation. Experimental results show that the formed PSBP PBG device can be spatially tuned from the blue (481.9 nm) to red (646.9 nm) regions within 14 mm at room temperature. The total tunable spectral range is 165 nm. In addition to the spatially tunable PBG feature, this study also discusses the tuning features of the PSDDBP laser. Experimental results indicate that the tunable band of the laser is 57.7 nm at room temperature, which is 82 nm narrower than that of the corresponding PBG. Three key factors lead to this narrowing: the dye’s reabsorption of fluorescence photons at short wavelength regions and weak dye’s fluorescence emission and significant fragmentation of the frustrated BP structure at long wavelength regions. The temperature sensitivity of the lasing wavelength for the PSDDBP laser is linear and approximately −0.26 nm/°C. This temperature sensitivity is primarily attributed to the constant and negative *dn/dT* of the LCs. Considering the advantages of the two devices, they have great potential for use in applications of photonic devices and displays. Nowadays, three-dimensional direct laser writing (3D DLW) lithography is being a powerful technique and widely employed to fabricate delicate 3D structures and photonic devices at microscale^36^,^37^,^38^,^39^. Therefore, the integration of 3D DLW lithography and the PSBP introduced in this work can open some new prospects, such as the miniaturization of the PSBP rainbow-like structure and the developments of tunable 3D micro PSBP photonic devices with free-form structures.

## Methods

### Sample preparation

To obtain a PSBP sample with a large pitch gradient, two steps are performed. The first step prepares two various BP-monomer mixtures including a low and a high chiral concentrations, namely, BP-monomer mixtures A and B, respectively. Table 1 indicates their prescriptions. The BP-monomer mixtures A (B) is formed by uniformly mixing 57.7 (55.7) *wt*% NLC (HTW11420-100, from Fusol Material), 30.0 (29.0) *wt*% chiral dopant 1 and 0.5 (3.5) *wt*% chiral dopant 2 (S811 and S1011, respectively, both from Fusol Material), 6.0 *wt*% diacrylate monomer (RM257, from Merck), 5.0 *wt*% 2,2-Bis[(acryloyloxy)methyl]butyl acrylate (TMPTA, from Sigma-Aldrich), and 0.8 *wt*% 2,2-dimethoxy-2-phenyl acetophenone (DMPAP, from Acros Organics). In HTW114200-100 NLC host, the HTP values of S811 and S1011 are about –11.24 μm^−1^ and –33.72 μm^−1^, respectively. The two kinds of achiral monomer, RM257 and TMPTA, are used to form polymer networks which may stabilize the BP structure. The diacrylate monomer, RM257, is used as a crosslinker for 3D network formation^40^. The triacrylate monomer, TMPTA, is used to form polymer network with high crosslinking density after polymerization because it contains three acrylate groups (C=C bonds) as the reactive sites^41^. In addition, TMPTA has a fast curing response, as mentioned in ref. 42. Because BPs are very sensitive to temperature, the disturbance caused by the heat generated via UV exposure can be minimized if the curing time could be reduced. This is helpful to the polymerization under a steady BP structure.

Two spacers of 15-μm-thickness are placed at the two edges of longer sides of two overlapped cleaned ITO glass slides. A longer spacer of same thickness is placed at the middle of the glass substrates. A glue is then used to seal the overlapped glasses at their two edges of longer sides to form the empty cell. After injecting the BP-monomer mixtures A and B into the empty cell with a middle spacer from the left and right sides, respectively, and then slowly drawing away the middle spacer from the sample, the two mixtures can reversely diffuse to each other and form a large pitch gradient along the diffusion direction in dark for around 7 days. This large pitch gradient may guarantee a large gradient distribution of PBG in the gradient-pitched BP-monomer sample. The temperature of the cell is slowly decreased from isotropic phase (30.5 °C) to 18.5 °C at a rate of 0.03 °C/min. Then, the cell is exposed with UV irradiation (peak wavelength: 365 nm) with an intensity of 1.1 mW/cm^2^ and a curing time of 30 minutes to form the gradient-pitched PSBP.

Furthermore, a laser dye (P597, from Exciton) with identical concentration (0.3 *wt*%) is individually added in the BP-monomer mixtures A and B to obtain the DDBP-monomer mixtures A and B, respectively. A gradient-pitched DDBP-monomer sample is instead obtained by replacing the BP-monomers A and B with DDBP-monomers A and B, respectively, and undergoing the same fabrication process described above. However, the laser dyes exposed to UV irradiation may transit to the excited state and then relax to the ground state via non-radiative transition^43^, resulting in the release of heat, and thus the increase of temperature. The significant heating generated by the strong and continuous UV irradiation on the laser dyes may disturb the orientation of the liquid crystal molecules in the double twist cylinders of the BP structure, and then induce lattice distortion and even phase transition from BP to isotropic state^16^,^17^,^44^. Under this condition, no BP structure can be formed after the UV-induced photopolymerization. To avoid the occurrence of this issue, this work adopts a low intensity UV irradiation (0.15 mW/cm^2^) and an intermittent curing method in which the gradient-pitched DDBP-monomer sample is irradiated for 30 s and unirradiated for 30 s intermittently, until the total curing time reaches 30 minutes. A gradient-pitched PSDDBP is then obtained.

### Experimental setups

An R-POM (IX71, Olympus) is the main tool for observing the reflective textures and measuring the reflectance of the gradient-pitched PSBP sample. The incident white light is focused on the sample which is pre-setup on a hot stage (TP102V, Instec) via an objective (20 × /NA = 0.45, LUC Plan FLN, Olympus) after going through a polarizer. The reflected light could be collected by the same objective and enter: (1) the spectrometer (Jaz-combo-2, resolution: ~0.9 nm, Ocean Optics) or (2) CCD (4000AM, DVC), after going through an analyzer. The PSBP sample is horizontally fixed on a translation stage, thus the spatial distribution of the reflection spectra and POM images of the sample could be obtained by choosing the different positions on the cell surface along the translational axis (x axis). The R-POM is also employed for observing the Kossel diagrams of the PSBP samples. A band-pass filter is placed in front of the white light source for generating a monochromatic light (with different wavelength according to requirement). In addition, a high NA oil immersion objective (×100/NA = 1.25, oil, Plan, Olympus) is attached on the microscope for converging the incident light, collecting the reflected light from the crystal planes of the PSBP samples, and projecting onto the back focal plan of the objective to form the Kossel diagrams^28^. Bertrand lens is not used in our setup because the focal length of the CCD camera (XCD-V60CR, SONY) is adjustable to capture the images on the back focal plane of the objective, that is, the Kossel diagrams.

For measuring the spatially tunable lasing emission from the PSDDBP sample, the PSDDBP sample is installed on a hot stage which is fixed on a XYZ translation stage. One Nd-YAG pulse laser (wavelength: 532 nm, repetition rate: 10 Hz, pulse duration: 8 ns, LAB-130-10, Spectra-Physics) is used as a pump source for exciting the lasing emission. The pumped energy of the incident pulses is adjusted by the combination of a half-wave plate and a polarizer. The incident pumped pulses beam is divided into two sub-beams with identical energy by a nonpolarizing beam splitter (BS). The reflected sub-beam is focused by a lens (focal length *f* = 10 cm) on the cell at an included angle of 27° from the normal direction of the sample surface, while the transmitted one is detected by an energy meter (1916C, Newport) for measuring the incident pumped energy. The lasing emission is detected along the normal direction. The continuously spatial tunability of the lasing peak of the PSDDBP can be measured by continuously moving the pumped position along x axis on the cell. The experimental setup for measuring the lasing emission spectra of the PSDDBP is shown in Supplementary Fig. S12.

## Additional Information

**How to cite this article**: Lin, J.-D. *et al*. Wide-Band Spatially Tunable Photonic Bandgap in Visible Spectral Range and Laser based on a Polymer Stabilized Blue Phase. *Sci. Rep.* **6**, 30407; doi: 10.1038/srep30407 (2016).

## Supplementary Material

Supplementary Information

## Figures and Tables

**Table 1:** Prescriptions for BP-monomer mixtures A and B.

Materials	NLC	Chiral dopant 1	Chiral dopant 2	Achiral monomer 1	Achiral monomer 2	Photoinitiator
	HTW 114200-100	S811	S1011	RM257	TMPTA	DMPAP
BP-monomer mixture A	57.7 *wt*%	30 *wt*%	0.5 *wt*%	6 *wt*%	5 *wt*%	0.8 *wt*%
BP-monomer mixture B	55.7 *wt*%	29 *wt*%	3.5 *wt*%	6 *wt*%	5 *wt*%	0.8 *wt*%
