# Supplementary material for: Wide-Band Spatially Tunable Photonic Bandgap in Visible Spectral Range and Laser based on a Polymer Stabilized Blue Phase
Source: Sci Rep. 2016 Jul 26;6:30407. doi: 10.1038/srep30407 (PMC4960656; doi:10.1038/srep30407)
Supplement: Supplementary Information [file srep30407-s1.doc]

**Wide-Band Spatially Tunable Photonic Bandgap in Visible Spectral Range and Laser based on a Polymer Stabilized Blue Phase**

**Jia-De Lin**^1,+^**, Tsai-Yen Wang**^1,+^**, Ting-Shan Mo**^2^**, Shuan-Yu Huang**^3,4^**, and Chia-Rong Lee**^1,5*^

^1^National Cheng Kung University, Department of Photonics, Tainan, 701, Taiwan

^2^Kun Shan University of Technology, Department of Electro-Optical Engineering, Tainan, 710, Taiwan

^3^Chung Shan Medical University, Department of Optometry, Taichung, 402, Taiwan

^4^Chung Shan Medical University Hospital, Department of Ophthalmology, Taichung, 402, Taiwan

5 National Cheng Kung University, Advanced Optoelectronics Technology Center, Tainan, 701, Taiwan

*[crlee@mail.](mailto:corresponding.author@email.example)ncku.edu.tw

+these authors contributed equally to this work

**Supplementary information**

**Temperature dependent colors of the BP-monomer mixtures**

Two BP-monomer mixtures A and B is employed in this work for the formation of a gradient-pitched PSBP sample. The reflective colors of the mixtures are dependent on temperature, as displayed in the R-POM images (Refer to Figs. S1 and S2).

**Figure S1.** Reflective POM images of cells with BP-monomer mixture A (B) at temperatures decreasing from (a) 26.5 °C to (j) 17.5 °C [from (k) 24.5 °C to (t) 15.5 °C].


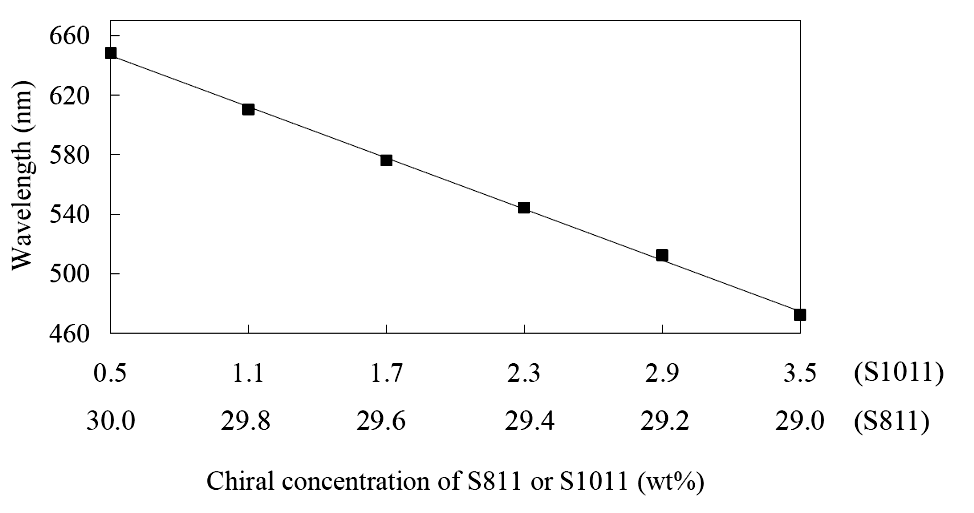


**Figure S2.** Variation of the peak wavelength of the PBG with the chiral concentration of S1011 or S811 for cells filling with BP-monomer mixtures A, C, D, E, F, and B at 18.5 °C.

**Kossel diagrams of PSBP cells**

Kossel diagram is a power technique to identify the crystal plane of a BP cell. The Kossel diagram is the reflected image of the observed BP crystal on the back focal plane of the objective lens of a reflection microscopy equipped with a monochromatic source. For obtaining Kossel lines completely, two conditions should be satisfied. One is that the incident wavelength of a monochromatic probe beam has to be shorter than the Bragg wavelength of normal reflecting light of these specific lattice planes, while the other one is that the NA value of the objective lens should be as high as possible. In this work, an oil immersion objective lens with NA value of 1.25 is employed. The incident wavelength of the monochromatic probe beam is determined by band-pass filters with central wavelength of 456, 488, 520, 535, 550, 580, and 610 nm, respectively. As shown in Fig. S3, incident light with above-mentioned wavelengths can be employed to obtain Kossel diagrams since all of they are shorter than the Bragg wavelength of normal reflecting light, 640 nm (Refer to Fig. S3).

Figures S4 and S5 display the R-POM images and the Kossel diagrams of PSBP cells C and D, respectively. The Kossel diagram of PSBP cell C degenerates to a point when the incident wavelength is 610 nm, as shown in Fig. S4(h). The degeneration of Kossel diagram is resulted from that the incident wavelength (610 nm) nearly corresponds to the Bragg wavelength of normal reflecting light of the PSBP (608 nm). The similar phenomenon can be seen in PSBP cell D when the incident wavelength is 580 nm, as shown in Fig. S5(g). If the incident wavelength is larger than the Bragg wavelength of normal reflecting light of a PSBP, no Kossel diagram but only background scattering can be observed, as shown in Figs. S5(h), S6(g), S6(h), S7(f)-S7(h), and S8(c)-S8(h), respectively (Refer to Figs. S4-S8).

**Figure S3.** (a) BP pattern observed under reflective POM and (b)−(h) measured Kossel diagrams of PSBP cell A detected by probe beams with wavelengths of λ =456, 488, 520, 535, 550, 580, and 610 nm, respectively. The normal reflection peak wavelength of the PBG in the cell is *λ_h,k,l(norm.)_* = 640 nm.

**Figure S4.** (a) BP pattern observed under reflective POM and (b)−(h) measured Kossel diagrams of PSBP cell C detected by probe beams with wavelengths of λ =456, 488, 520, 535, 550, 580, and 610 nm, respectively. The normal reflection peak wavelength of the PBG in the cell is *λ_h,k,l(norm.)_* = 608 nm.

**Figure S5.** (a) BP pattern observed under reflective POM and (b)−(h) measured Kossel diagrams of PSBP cell D detected by probe beams with wavelengths of λ =456, 488, 520, 535, 550, 580, and 610 nm, respectively. The normal reflection peak wavelength of the PBG in the cell is *λ_h,k,l(norm.)_* = 576 nm.

**Figure S6.** (a) BP pattern observed under reflective POM and (b)−(h) measured Kossel diagrams of PSBP cell E detected by probe beams with wavelengths of λ =456, 488, 520, 535, 550, 580, and 610 nm, respectively. The normal reflection peak wavelength of the PBG in the cell is *λ_h,k,l(norm.)_* = 540 nm.

**Figure S7.** (a) BP pattern observed under reflective POM and (b)−(h) measured Kossel diagrams of PSBP cell F detected by probe beams with wavelengths of λ =456, 488, 520, 535, 550, 580, and 610 nm, respectively. The normal reflection peak wavelength of the PBG in the cell is *λ_h,k,l(norm.)_* = 512 nm.

**Figure S8.** (a) BP pattern observed under reflective POM and (b)−(h) measured Kossel diagrams of PSBP cell B detected by probe beams with wavelengths of λ =456, 488, 520, 535, 550, 580, and 610 nm, respectively. The normal reflection peak wavelength of the PBG in the cell is *λ_h,k,l(norm.)_* = 470 nm.

**Spatially-tunable gradient-pitched PSDDBP lasers**

The gradient-pitched PSDDBP cell shows a rainbow-like reflection pattern from *x* = 0 mm to 14 mm. The grain-like reflective appearance with various colors in the cell (Fig. S9) is attributable to the selective reflection of the incident light from the micro-platelets of the BP at various positions of the cell (Fig. S10).

**Figure S9.** Reflective appearance of the formed gradient-pitched PSDDBP sample.

**Figure S10.** (a)-(h) Reflective POM images of the gradient-pitched PSDDBP sample at *x* = 0, 2, 4, 6, 8, 10, 12, and 14 mm, respectively. Scale bar presents 100 μm.

**Lasing features of the gradient-pitched PSDDBP laser at different pumped positions**

The energy threshold of the gradient-pitched PSDDBP laser at different pumped positions can be determined by plotting the variations of the lasing intensity and the corresponding FWHM with the pumped energy, as shown in Fig. S11. The energy threshold is the pumped energy above which the emission intensity increases abruptly and the FWHM narrows suddenly (Refer to Fig. S11).


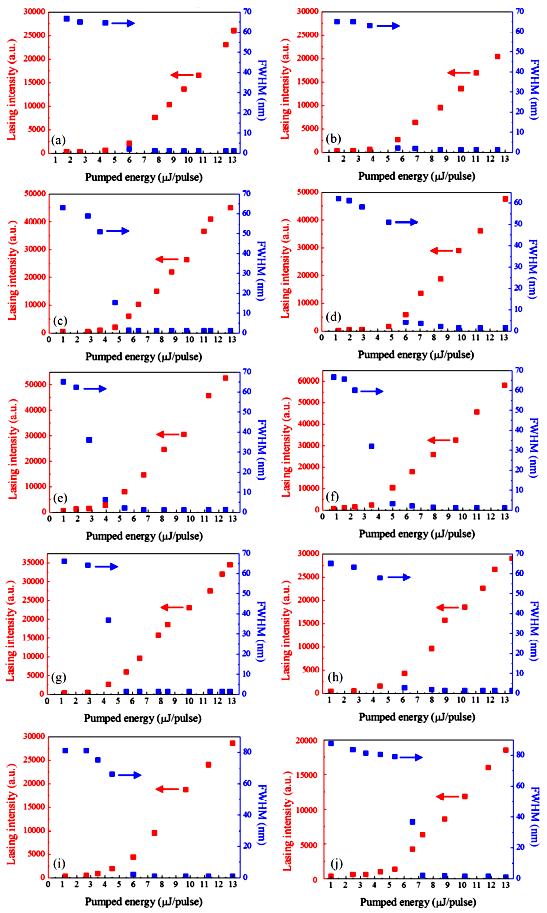

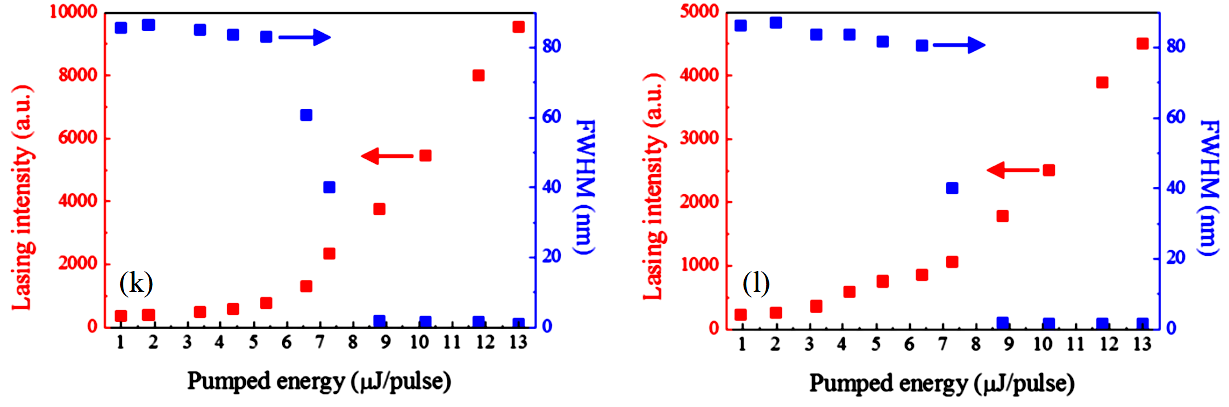


**Figure S11.** (a) − (l) Variations of the lasing intensity and the corresponding FWHM of the gradient-pitched PSDDBP laser with the pumped energy at pumped positions of *x* = 2.0 − 7.5 mm, respectively, with a decreasing pumped step of 0.5 mm.

**Experimental setup in lasing examination based on the gradient-pitched PSDDBP sample**

For measuring the spatially tunable lasing emission of the PSDDBP cell, the PSDDBP cell is installed on a hot stage which is fixed on a XYZ translation stage. One Nd-YAG pulse laser (wavelength: 532 nm, repetition rate: 10 Hz, pulse duration: 8 ns, LAB-130-10, Spectra-Physics) is used as a pump source for generating the lasing emission. The pumped energy of the incident pulses can be controlled by the combination of a half-wave plate and a polarizer. The incident pumped pulses beam is divided into two sub-beams with identical energy by a nonpolarizing beam splitter (BS). The reflected sub-beam is focused by a lens (focal length *f* = 10 cm) on the cell at an included angle of 27° from the normal direction of the sample surface, while the transmitted one is detected by an energy meter (1916C, Newport) for measuring the incident pumped energy. The lasing emission is detected along the normal direction. The continuously spatial tunability of the lasing peak of the PSDDBP can be measured by continuously moving the pumped position along x axis on the cell.


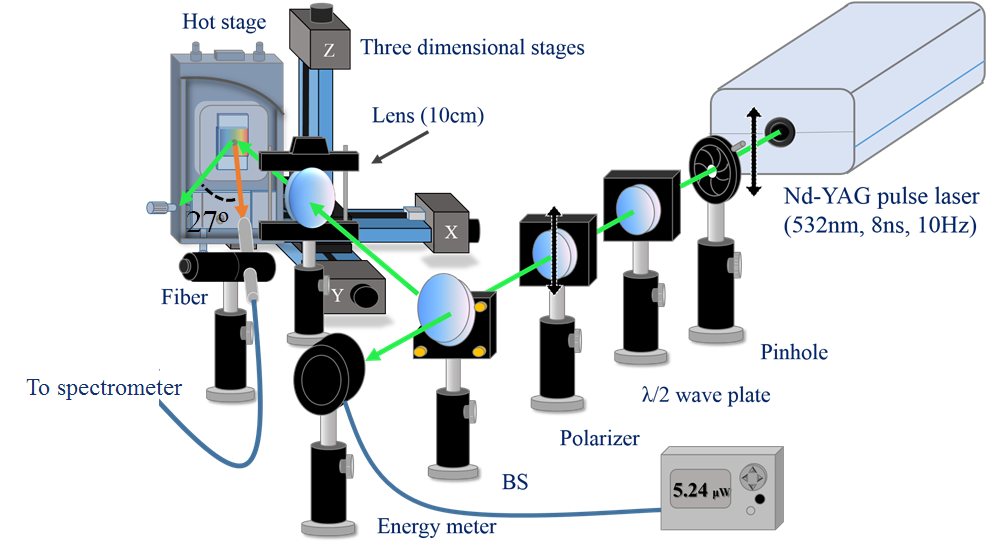


**Figure S12.** Experimental setup for measuring the lasing emission spectra of the PSDDBP.


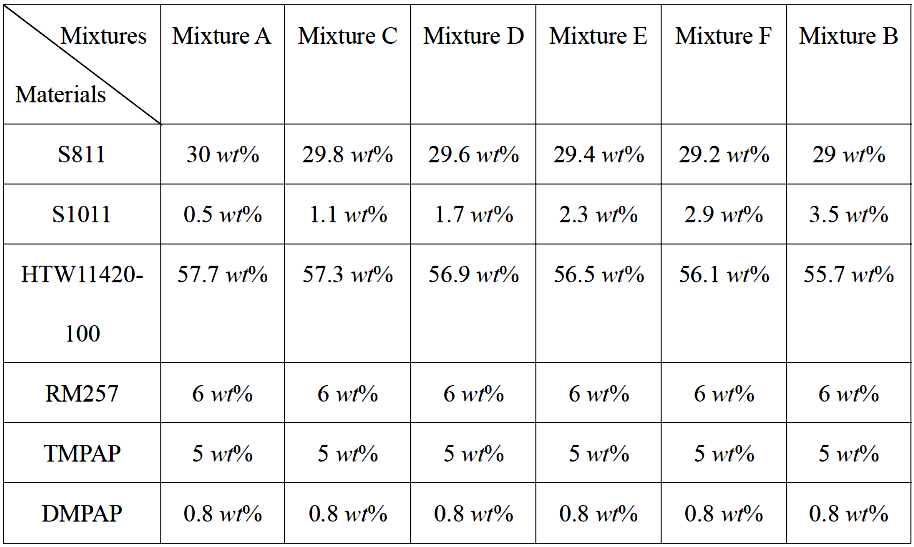


**Table S1.** Chiral contains of BP-monomer mixtures A, C, D, E, F, and B. The concentrations of the monomers and photoinitiator for all mixtures are designed to be the same.

**
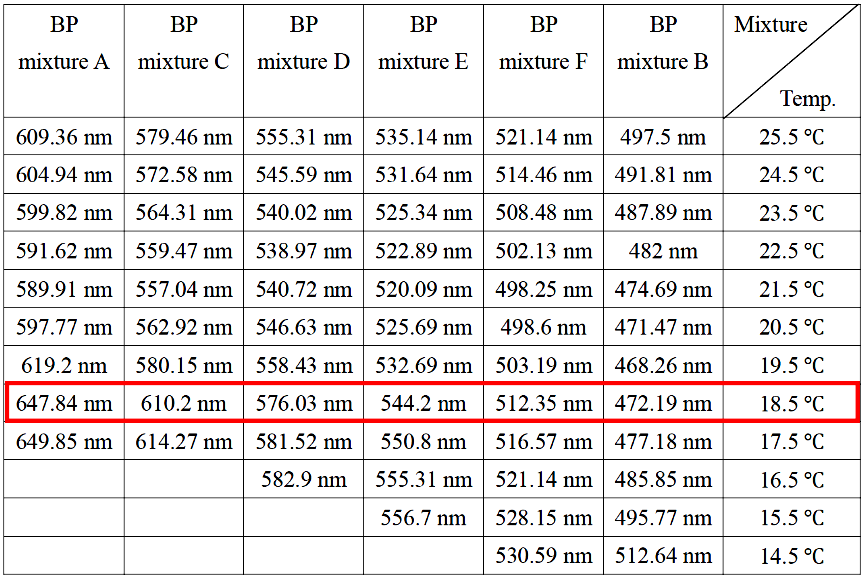
**

**Table S2.** Variations of reflection peak wavelength with temperature for BP-monomer mixtures A, C, D, E, F, and B.
